# Algorithm for diagnosing hypertension using out‐of‐office blood pressure measurements

**DOI:** 10.1111/jch.14382

**Published:** 2021-10-26

**Authors:** Je Sang Kim, Moo‐Yong Rhee, Chee Hae Kim, Yoo Ri Kim, Ungjeong Do, Ji‐Hyun Kim, Young Kwon Kim, Hyun Jung Lee, Jee Yeon Park, June Namgung, Sung Yun Lee, Deok‐Kyu Cho, Tae‐Young Choi, Seok Yeon Kim

**Affiliations:** ^1^ Cardiovascular Center Dongguk University Ilsan Hospital Goyang South Korea; ^2^ Division of Hematology and Medical Oncology Dongguk University Ilsan Hospital Goyang South Korea; ^3^ Department of Internal Medicine Dongguk University Ilsan Hospital Goyang South Korea; ^4^ Division of Cardiology Department of Internal Medicine Ilsan Paik Hospital Inje University College of Medicine Goyang South Korea; ^5^ Division of Cardiology Yongin Severance Hospital Yonsei University College of Medicine Yongin South Korea; ^6^ Department of Internal Medicine Seoul Red Cross Hospital Seoul South Korea; ^7^ Department of Internal Medicine Seoul Medical Center Seoul South Korea

**Keywords:** algorithm, hypertension, out‐of‐office blood pressure

## Abstract

The authors developed and validated a diagnostic algorithm using the optimal upper and lower cut‐off values of office and home BP at which ambulatory BP measurements need to be applied. Patients presenting with high BP (≥140/90 mm Hg) at the outpatient clinic were referred to measure office, home, and ambulatory BP. Office and home BP were divided into hypertension, intermediate (requiring diagnosis using ambulatory BP), and normotension zones. The upper and lower BP cut‐off levels of intermediate zone were determined corresponding to a level of 95% specificity and 95% sensitivity for detecting daytime ambulatory hypertension by using the receiver operator characteristic curve. A diagnostic algorithm using three methods, OBP‐ABP: office BP measurement and subsequent ambulatory BP measurements if office BP is intermediate zone; OBP‐HBP‐ABP: office BP, subsequent home BP measurement if office BP is within intermediate zone and subsequent ambulatory BP measurement if home BP is within intermediate zone; and HBP‐ABP: home BP measurement and subsequent ambulatory BP measurements if home BP is within intermediate zone, were developed and validated. In the development population (*n* = 256), the developed algorithm yielded better diagnostic accuracies than 75.8% (95%CI 70.1–80.9) for office BP alone and 76.2% (95%CI 70.5–81.3) for home BP alone as follows: 96.5% (95%CI: 93.4–98.4) for OBP‐ABP, 93.4% (95%CI: 89.6–96.1) for OBP‐HBP‐ABP, and 94.9% (95%CI: 91.5–97.3%) for HBP‐ABP.  In the validation population (*n* = 399), the developed algorithm showed similarly improved diagnostic accuracy. The developed algorithm applying ambulatory BP measurement to the intermediate zone of office and home BP improves the diagnostic accuracy for hypertension.

## 1 INTRODUCTION

Compared to office blood pressure (BP), out‐of‐office BP has demonstrated better predictive power for hypertension‐mediated organ damage and cardiovascular events.^1^, ^2^, ^3^, ^4^ Additionally, diagnosis and treatment of hypertension solely using office BP measurements possess an inherent risk of over‐treatment of white‐coat hypertension or under‐treatment of masked hypertension. Therefore, recent guidelines recommend greater application of out‐of‐office BP measurements in the diagnosis and treatment of hypertension, particularly when white‐coat or masked hypertension is suspected.^5^, ^6^

Ambulatory BP measurement is preferred to home BP measurement because ambulatory BP provides better cardiovascular risk prediction than home BP.^3^, ^7^ However, ambulatory BP measurement is not conducted for all patients because of high cost and inconvenience. Alternatively, home BP could be easily measured; however, ambulatory and home BP measurement methods are not interchangeable because there is a diagnostic disagreement between these two methods.^8^, ^9^, ^10^, ^11^ It is impractical to measure ambulatory or home BP in all patients, and it is not easy to determine which method could be most appropriately applied for the most suitable patients.

Several algorithms for the diagnosis of hypertension using ambulatory and home BP measurements have been proposed.^12^, ^13^, ^14^, ^15^, ^16^, ^17^ However, most studies focused on diagnosing white‐coat hypertension, not on masked hypertension, and did not apply all available office, home, and ambulatory BP measurement methods. Moreover, existing guidelines do not clearly indicate the patients most suitable for ambulatory and home BP measurement for diagnosing hypertension,^5^, ^6^ and both are overused.^17^, ^18^ Although stage 1 hypertension and high normal BP were suggested as conditions in which out‐of‐office BP measurements are required,^5^, ^6^ the upper and lower BP thresholds, which necessitate out‐of‐office BP measurement to distinguish white‐coat and masked hypertension from sustained hypertension and normotension, have not been adequately investigated. Therefore, practitioners are easily confused in determining patients where ambulatory and home BP measurements are appropriate.

In this study, we developed and validated a diagnostic algorithm by determining the optimal upper and lower cut‐off BP levels at which ambulatory or home BP measurement need to be applied.

## 2 METHODS

### 2.1 Study population

The diagnostic algorithm was developed using data from the study conducted between March 2012 and September 2013,^8^, ^19^ and validated with data of the study conducted between January 2015 and December 2019 (ClinicalTrials.gov identifier: NCT03855605).^11^ The study of the development population was conducted at four clinical trial centers in Korea (Dongguk University Ilsan Hospital, Inje University Ilsan Paik Hospital, Myongji Hospital, and Seoul Medical Center), and the study of the validation population was conducted at a single center (Dongguk University Ilsan Hospital).

The inclusion criteria were the same for the two studies. Individuals who had high office BP (≥ 140/90 mm Hg) at the outpatient clinic, were not being managed on antihypertensive drugs, needed a diagnosis of hypertension, and were aged ≥ 20 years were prospectively enrolled in the studies. Individuals with secondary hypertension; hypertensive emergency or urgency; heart failure (New York Heart Association class III and IV); clinically significant cardiac arrhythmia; impaired renal function (serum creatinine ≥ 1.7 mg/dl); pregnancy; participating in night labor or shift work; history of drug or alcohol abuse within 6 months; current participation in other clinical studies; taking other clinical trial drugs within the past month; and taking drugs known to affect BP, such as steroids, monoamine oxidase inhibitors, oral contraceptives, or sympathomimetics, were excluded. The study protocols and informed consent were reviewed and approved by the Institutional Review Board of participating hospitals. All participants provided written informed consent before entry into the study.

### 2.2 Measurements for office, home, and ambulatory BP

BP measurement schedule (Figure S1 in the supplementary material) was also described elsewhere,^8^, ^11^, ^19^ and it did not differ significantly between the studies.

Office BP was measured by the study nurses on the first visit day, second visit day after completion of the home BP measurement, and on the third visit day after the completion of 24‐h ambulatory BP measurement. Office BP was measured with validated oscillometric devices (WatchBP Home; Microlife, Taiwan for development population and WatchBP Office; Microlife, Taiwan for validation population). Participants were asked to avoid smoking, caffeine‐containing beverages, and exercise within 30 min preceding the measurements. Three readings of office BP after 5 min of seated rest in a quiet room and at 1‐min intervals were obtained at each visit using an appropriate cuff size. In the development study population, the office BP were measured trice from both arms on the first visit, and the arm with higher BP was determined and designated as the index arm. During the second and third visits, office BP was measured from the index arm. In the validation population, office BP was measured from both arms simultaneously three times during every three visits. The BPs of both arms were averaged, and the BP of the arm with the higher averaged BP was used as the office BP of the index arm.

Home BP was measured with validated oscillometric device (WatchBP Home; Microlife, Taiwan) in both studies. Participants were instructed to take triplicate measurements at 1 min intervals every morning (between 07:00 h or waking and 09:00 h) and every evening (between 21:00 h and 23:00 h or before bedtime) for seven consecutive days. In the validation study, home BP measurements for 9 days were allowed if the participants desired. Participants were instructed to measure BP in the morning after micturition or defecation, and before showering and breakfast. On the last day of home BP measurement, participants measured the last morning home BP and visited the clinical trial centers (second visit). Based on our previous findings, a valid measurement of home BP was defined as at least 5 days of morning and evening duplicate measurements.^19^ The home BP measured on the evening of the first day and on the morning of the second day were discarded, and the first and second readings of each session were averaged (the third reading was discarded).^19^

At the second visit, ambulatory BP monitoring over 25 h was performed on the non‐dominant arm using an automated, noninvasive oscillometric device (Mobil‐O‐Graph, I.E.M GmbH, Germany), with a measurement interval of 30 min. Participants were instructed to continue normal daily activities during the day. A valid measurement was defined as valid readings for > 70% of the total measurement attempts, and at least 14 measurements during the daytime (10:00 to 20:00 h for development population, 09:00 to 21:00 for validation population) and at least seven measurements during the nighttime (00:00 to 06:00 h).

A blood sample for hematologic and biochemical analysis was obtained after at least 8 h of overnight fasting.

### 2.3 Definition of hypertension

The mean daytime ambulatory BP was used as a reference standard for the diagnosis of hypertension. Average daytime systolic BP (SBP) ≥135 mm Hg and/or diastolic BP (DBP) ≥85 mm Hg were defined as hypertension. Office BP hypertension was defined as office SBP ≥140 mm Hg and/or DBP ≥90 mm Hg. Home BP hypertension was defined as average home SBP ≥135 mm Hg and/or DBP ≥85 mm Hg.

White‐coat hypertension using office BP was defined as meeting criteria for office BP hypertension but having normal daytime ambulatory BP. Masked hypertension using office BP was defined as meeting criteria for office BP normotension but having daytime ABP hypertension. White‐coat and masked hypertension using home BP was defined as meeting criteria for home BP hypertension but having normal daytime ambulatory BP and home BP normotension but having daytime ABP hypertension, respectively.

### 2.4 Statistical analysis

SBP and DBP for office and home BP were divided into three categories: the hypertension zone and normotension zone, not requiring diagnosis by ambulatory BP measurement, and the intermediate zone requiring diagnosis using ambulatory BP measurement. To identify upper and lower cut‐off levels of SBP and DBP for office and home BP, the receiver operator characteristic (ROC) curve was used. The upper boundary of SBP and DBP was determined corresponding to a level of 95% specificity for detecting ambulatory hypertension and the lower boundary corresponding to a level of 95% sensitivity for detecting ambulatory hypertension. For the practicality and convenience of algorithm application, we determined the nearest value of the multiples of 5 mm Hg as the cut‐off value of the intermediate zone, in case the cut‐off values were located between the multiples of 5 mm Hg (eg, 130, 135, 140, and 145 mm Hg).

In the development of the algorithm, we created three methods: (1) diagnosis of hypertension by office BP measurements for three visits and additional ambulatory BP measurements in the intermediate zone of office BP (OBP‐ABP method), (2) diagnosis of hypertension by office BP measurements for 3 days, additional home BP measurement in the intermediate zone of office BP, and additional ambulatory BP measurements in the intermediate zone of home BP (OBP‐HBP‐ABP method), and (3) diagnosis of hypertension by home BP measurements and additional ambulatory BP measurement in the intermediate zone of home BP (HBP‐ABP method).

The diagnostic sensitivity, specificity, positive predictive value, and negative predictive value of office BP and home BP measurement referenced to the diagnosis of hypertension by daytime ambulatory BP were analyzed for all participants and participants excluding intermediate zone in the development and validation population. The diagnostic sensitivity, specificity, positive predictive value, negative predictive value, and accuracy (Table S1 in the supplementary material) of each method were analyzed in the development and validation population. In the comparison of diagnostic accuracies, non‐overlapping of 95% confidence intervals indicates statistical difference.

MedCalc software, version 19.6.4 (MedCalc Software bvba; Ostend, Belgium) was used for statistical analyses.

## 3 RESULTS

### 3.1 Characteristics of study population

In development of the algorithm, 319 participants were recruited, and the data of 256 participants (mean age: 51.8±9.7 years; men: 119) with valid home BP and 24‐h ambulatory BP measurements were analyzed. In the validation population, 470 participants were recruited, and data of 399 participants (mean age: 52.4±9.8 years; men: 126) with valid home BP and 24‐h ambulatory BP measurements were analyzed. The baseline clinical characteristics of the development and validation population are summarized in Table 1. There was no difference in mean age and ratio of sexes between development and validation populations. The validation population showed a significantly higher prevalence of diabetes, drinkers, level of daytime SBP, and home SBP. The prevalence of white‐coat and masked hypertension using office BP between development and validation populations was similar (7.8% vs. 7.3%, *p* = 0.813, and 16.4% vs. 15.5% *p* = 0.759, respectively).

**TABLE 1:** Baseline characteristics and blood pressure of study population

	Development (*n* = 256)	Validation (*n* = 399)	*p* value
Age (years)	51.6 ± 9.7	52.5 ± 10.3	.295
Sex (male), *n* (%)	120 (46.9)	192 (48.1)	.756
Body mass index (kg/m^2^)	25.4±3.4	25.3±3.4	.549
Diabetes mellitus, *n* (%)	8 (3.1)	33 (8.3)	.008
Current smoking, *n* (%)	48 (18.7)	63 (15.8)	<.001
Alcohol use, *n* (%)	126 (49.2)	241 (60.4)	.005
Office systolic BP (mm Hg)	141.1±12.6	141.8±10.4	.273
Office diastolic BP (mm Hg)	91.6±9.6	91.9±8.9	.584
Home systolic BP (mm Hg)	134.6±13.6	136.8±11.9	.033
Home diastolic BP (mm Hg)	87.8±9.9	88.2±9.3	.588
24 h ambulatory systolic BP (mm Hg)	132.8±12.4	134.6±12.1	.070
24 h ambulatory diastolic BP (mm Hg)	88.5±11.2	88.9±10.5	.605
Daytime ambulatory systolic BP (mm Hg)	137.2±14.6	139.3±13.1.	.046
Daytime ambulatory diastolic BP (mm Hg)	91.9±12.5	92.8±11.2	.336
Hypertension phenotypes			
Normotension, *n* (%)	48 (18.8)	45 (11.3)	
White‐coat hypertension, *n* (%)	20 (7.8)	29 (7.3)	
Masked hypertension, *n* (%)	42 (16.4)	62 (15.5)	
Sustained hypertension, *n* (%)	146 (57.0)	263 (65.9)	

*Abbreviation*: BP, blood pressure.

### 3.2 Development of diagnostic algorithm

Office BP at 95% sensitivity for detecting ambulatory hypertension was 130.2 mm Hg for SBP and 81.1 mm Hg for DBP. Office BP at 95% specificity for detecting ambulatory hypertension was 146.1 mm Hg for SBP and 96.2 mm Hg for DBP. The three categories for office BP were determined: normotension zone < 130/80 mm Hg, intermediate zone 130–144/80–94 mm Hg, and hypertension zone ≥145/95 mm Hg. Home BP at 95% sensitivity for detecting ambulatory hypertension was 120.5 mm Hg for SBP and 78.0 mm Hg for DBP. Home BP at 95% specificity for detecting ambulatory hypertension was 143.7 mm Hg for SBP and 94.0 mm Hg for DBP. The three categories of home BP were determined: normotension < 120/80 mm Hg, intermediate 120–144/80–94 mm Hg, and hypertension zone ≥145/95 mm Hg.

In the intermediate zone of office BP, 65.0% of white‐coat hypertension and 95.2% of masked hypertension by office BP were included (Table 2). In the intermediate zone of home BP, 81.7% of white coat hypertension and 77.5% of masked hypertension by home BP were included (Table 2). In the intermediate zone of office BP and home BP, 85.0% of office BP white‐coat and masked hypertension and 78.7% of home BP white coat and masked hypertension were included.

**TABLE 2:** Distribution of hypertension phenotypes according to zones of office and home blood pressure values

	Office BP			Home BP		
	Normotension zone	Intermediate zone	Hypertension zone	Normotension zone	Intermediate zone	Hypertension zone
Development population						
NT, *n* (%)	14 (29.2)	34 (70.8)	0 (0.0)	24 (51.1)	23 (48.9)	0 (0.0)
WH, *n* (%)	0 (0.0)	13 (65.0)	7 (35.0)	0 (0.0)	17 (81.0)	4 (19.0)
MH, *n* (%)	2 (4.8)	40 (95.2)	0 (0.0)	9 (22.5)	31 (77.5)	0 (0.0)
SH, *n* (%)	0 (0.0)	38 (26.0)	108 (74.0)	0 (0.0)	76 (51.4)	72 (48.6)
Validation population						
NT, *n* (%)	12 (26.7)	33 (73.3)	0 (0.0)	10 (18.2)	45 (81.8)	0 (0.0)
WH, *n* (%)	0 (0.0)	19 (65.5)	10 (34.5)	0 (0.0)	18 (100.0)	0 (0.0)
MH, *n* (%)	4 (6.5)	58 (93.5)	0 (0.0)	10 (14.5)	59 (85.5)	0 (0.0)
SH, *n* (%)	0 (0.0)	88 (33.5)	175 (66.5)	0 (0.0)	140 (54.5)	117 (45.5)

Hypertension phenotypes in office BP and home BP were defined by office BP hypertension (≥140/90 mm Hg) and ambulatory BP hypertension (≥135/85 mm Hg), and home BP hypertension (≥135/85 mm Hg) and ambulatory daytime BP hypertension (≥135/85 mm Hg), respectively.

*Abbreviations*: BP, blood pressure; NT, normotension; WH, white‐coat hypertension; MH, masked hypertension; SH, sustained hypertension.

The diagnostic sensitivity and specificity of the diagnostic threshold of office BP 140/90 mm Hg were 77.7% (95% CI: 71.0–83.4%) and 70.6% (95% CI: 58.3–81.0%), and the diagnostic accuracy was 75.8% (95% CI: 70.1–80.9%) in the development populations. However, excluding individuals in the intermediate zone of office BP, the diagnostic sensitivity was improved to 98.2% (95% CI: 93.6–99.8%), and specificity was not changed (66.7%, 95% CI: 43.0–85.4%). The negative predictive value was improved (Table 3). The diagnostic sensitivity and specificity of diagnostic threshold of home BP 135/85 mm Hg were 78.7% (95% CI: 72.2–84.3%) and 69.1% (95%CI = 56.7–79.8%), and the diagnostic accuracy was 76.2% (95% CI: 70.5–81.3%) in the development population. Excluding individuals in the intermediate zone of home BP, the diagnostic sensitivity, specificity and accuracy were improved to 88.9% (95% CI: 80.0–94.8%), 85.7% (95% CI: 67.3–96.0%) and 88.1% (95% CI: 80.5–93.5%), respectively, but statistically insignificant (Table 3).

**TABLE 3:** Diagnostic accuracy of office blood pressure and home blood pressure based diagnosis of hypertension, referenced to daytime ambulatory blood pressure

	Development population					Validation population				
	Sensitivity, %(95%CI)	Specificity, % (95%CI)	PPV, % (95%CI)	NPV, % (95%CI)	Accuracy % (95%CI)	Sensitivity, % (95%CI)	Specificity, % (95%CI)	PPV, % (95%CI)	NPV, % (95%CI)	Accuracy % (95%CI)
All										
Office BP	77.7 (71.0–83.4)	70.6 (58.3–81.0)	88.0 (83.4–91.4)	53.3 (45.7–60.9)	75.8 (70.1–80.9)	80.7 (76.0–84.8)	61.6 (49.5–72.8)	90.4 (87.5–92.7)	41.7 (34.9–48.7)	77.2 (72.8–81.2)
Home BP	78.7 (72.2–84.3)	69.1 (56.7–79.8)	87.6 (83.1–91.0)	54.0 (46.1–61.7)	76.2 (70.5–81.3)	78.8 (74.0–83.1)	75.3 (63.9–84.7)	93.5 (90.5–95.5)	44.4 (38.4–50.5)	78.2 (73.8–82.2)
Excluding intermediate zone										
Office BP	98.2 (93.6–99.8)	66.7 (43.0–85.4)	93.9 (89.4–96.6)	87.5 (63.2–96.6)	93.1 (87.4 –96.8)	97.8 (94.4–99.4)	54.5 (32.2–75.6)	94.6 (91.7–96.5)	75.0 (51.4–89.5)	93.0 (88.6–96.1)
Home BP	88.9 (80.0–94.8)	85.7 (67.3–96.0)	94.7 (87.9–97.8)	72.7 (58.6–83.4)	88.1 (80.5–93.5)	92.1 (86.0–96.2)	100.0 (69.2–100.0)	100.0	50.0 (35.6–64.4)	92.7 (87.0–96.4)

Non‐overlapping of 95% confidence intervals indicates statistical difference.

*Abbreviations*: PPV, positive predictive value; NPV, negative predictive value; CI, confidence interval; BP, blood pressure.

#### 3.2.1 Office BP arm

**FIGURE 1:**
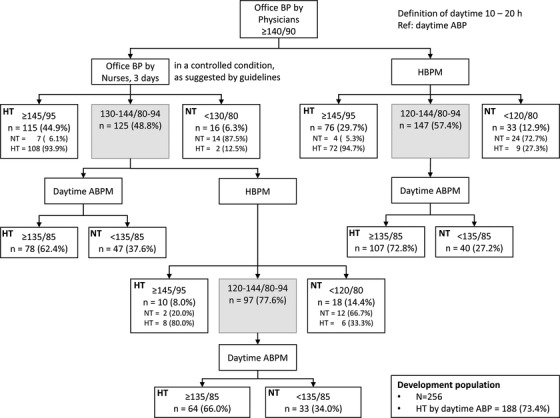
Distribution of participants based on the level of office blood pressure, home blood pressure, and ambulatory daytime blood pressure in the development population. BP: blood pressure, HT: hypertension, NT: normotension, ABP: ambulatory blood pressure, ABPM: ambulatory blood pressure measurements, HBPM: home blood pressure measurements

Among 256 participants, 115 (44.9%) individuals were in the hypertension zone, 125 (48.8%) in the intermediate zone, and 16 (6.3%) in the normotension zone of office BP (Figure 1). Among the individuals in the intermediate zone of office BP, 78 (62.4%) were determined as hypertension by daytime ambulatory BP (OBP‐ABP method). Among the individuals in the intermediate zone of office BP, 97 individuals (77.6%) were in the intermediate zone of home BP, 10 (8.0%) in the hypertension zone, and 18 (14.4%) in the normotension zone. Among the individuals in the intermediate zone of home BP, 64 (66.0%) were determined as hypertensive by daytime ambulatory BP (OBP‐HBP‐ABP method).

#### 3.2.2 Home BP arm

Among the participants, 76 individuals (29.7%) were in the hypertension zone, 147 (57.4%) in the intermediate zone, and 33 (12.9%) in the normotension zone of home BP (Figure 1). Among the individuals in the intermediate zone of home BP, 107 (72.8%) were determined as hypertension by daytime ambulatory BP (HBP‐ABP strategy).

#### 3.2.3 Diagnostic accuracy of algorithm

The diagnostic sensitivities and specificities of the three arms were more than 95% and 86%, respectively. AUCs were more than 0.9 (Table 4). The positive predictive values exceeded 95%. The negative predictive value of OBP‐ABP arm was 96.8% (95% CI: 88.5–99.2%) that is higher than those of OBP‐HBP‐ABP (88.1%, 95% CI:78.8–93.6%) and HBP‐ABP arms (87.7%, 95% CI: 78.9–93.1%), respectively, but statistically insignificant. The diagnostic accuracy was 96.5% (95% CI: 93.4–98.4) for the OBP‐ABP arm, 93.4% (95% CI: 89.6–96.1) for OBP‐HBP‐ABP arm, and 94.9% (95% CI: 91.5–97.3%) for HBP‐ABP arm, respectively.

**TABLE 4:** Diagnostic accuracy of three strategies in the development and validation population

	Development population			Validation population		
	OBP‐ABP	OBP‐HBP‐ABP	HBP‐ABP	OBP‐ABP	OBP‐HBP‐ABP	HBP‐ABP
Sensitivity, % (95% CI)	98.9 (96.2–99.9)	95.7 (91.8–98.1)	95.2 (91.1–97.8)	98.8 (96.9–99.7)	96.6 (94.0–98.3)	96.9 (94.4–98.5)
Specificity, % (95% CI)	89.7 (79.9–95.8)	86.8 (76.4–93.8)	94.1 (85.6–98.4)	86.3 (76.2–93.2)	86.3 (76.2–93.2)	100.0 (95.1–100.0)
AUC	0.943 (0.907–0.968)	0.913 (0.871–0.944)	0.947 (0.912–0.971)	0.925 (0.895–0.949)	0.915 (0.883–0.940)	0.985 (0.967–0.994)
PPV, % (95% CI)	96.4 (92.9–98.2)	95.2 (91.6–97.4)	97.8 (94.5–99.1)	97.0 (94.8–98.3)	96.9 (94.7–98.2)	100.0
NPV, % (95% CI)	96.8 (88.5–99.2)	88.1 (78.8–93.6)	87.7 (78.9–93.1)	94.0 (85.6–97.7)	85.1 (76.1–91.2)	88.0 (79.9–93.1)
Accuracy, % (95% CI)	96.5 (93.4–98.4)	93.4 (89.6–96.1)	94.9 (91.5–97.3)	96.5 (94.2–98.1)	94.7 (92.1–96.7)	97.5 (95.4–98.8)

Non‐overlapping of 95% confidence intervals indicates statistical difference.

*Abbreviations*: AUC, Area under the curve; PPV, positive predictive value; NPV, negative predictive value; CI, confidence interval.

### 3.3 Validation of diagnostic algorithm

The prevalence of white‐coat and masked hypertension in the intermediate zone were not different from those of the development population (Table 2). In the intermediate zone of office BP, 65.5% of white‐coat hypertension and 93.5% of masked hypertension by office BP were included. In the intermediate zone of home BP, 100.0% of white coat hypertension and 85.5% of masked hypertension by home BP were included. In the intermediate zone of office BP and home BP, 78.7% of office BP white‐coat and masked hypertension, and 91.7% of home BP white coat and masked hypertension were included.

**FIGURE 2:**
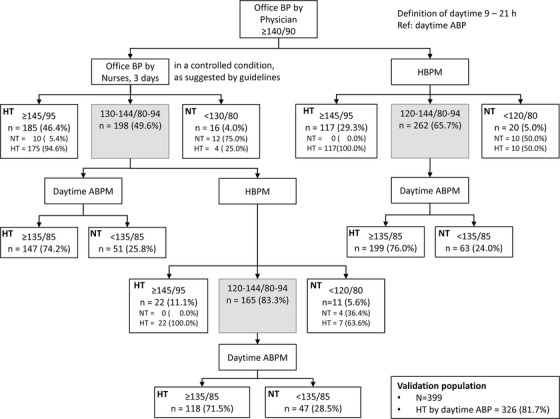
Distribution of participants based on the level of office blood pressure, home blood pressure, and ambulatory daytime blood pressure in the validation population. BP: blood pressure, HT: hypertension, NT: normotension, ABP: ambulatory blood pressure, ABPM: ambulatory blood pressure measurements, HBPM: home blood pressure measurements

The distribution of patients in the normotension, hypertension, and intermediate zones was similar to that in the development population (Figure 2). As in the development population, the diagnostic sensitivity was also improved significantly, excluding individuals in the intermediate zone of office and home BP (Table 3). The diagnostic sensitivity, specificity, and accuracies of each arm in the validation population were similar to those in the development population (Table 4).

### 3.4 Twenty‐four hour ambulatory BP as a reference standard for the diagnosis of hypertension

The algorithm using 24‐h ambulatory BP as a reference standard for the diagnosis of hypertension showed similar results with the algorithm using daytime ambulatory BP. The majority of masked and white coat hypertension was distributed in the intermediate zone, and diagnostic accuracy in the validation population also had good consistency to the development population, with accuracies exceeding 95% (Tables S2–S4, and Figures S2 and S3 in the supplementary material).

## 4 DISCUSSION

**FIGURE 3:**
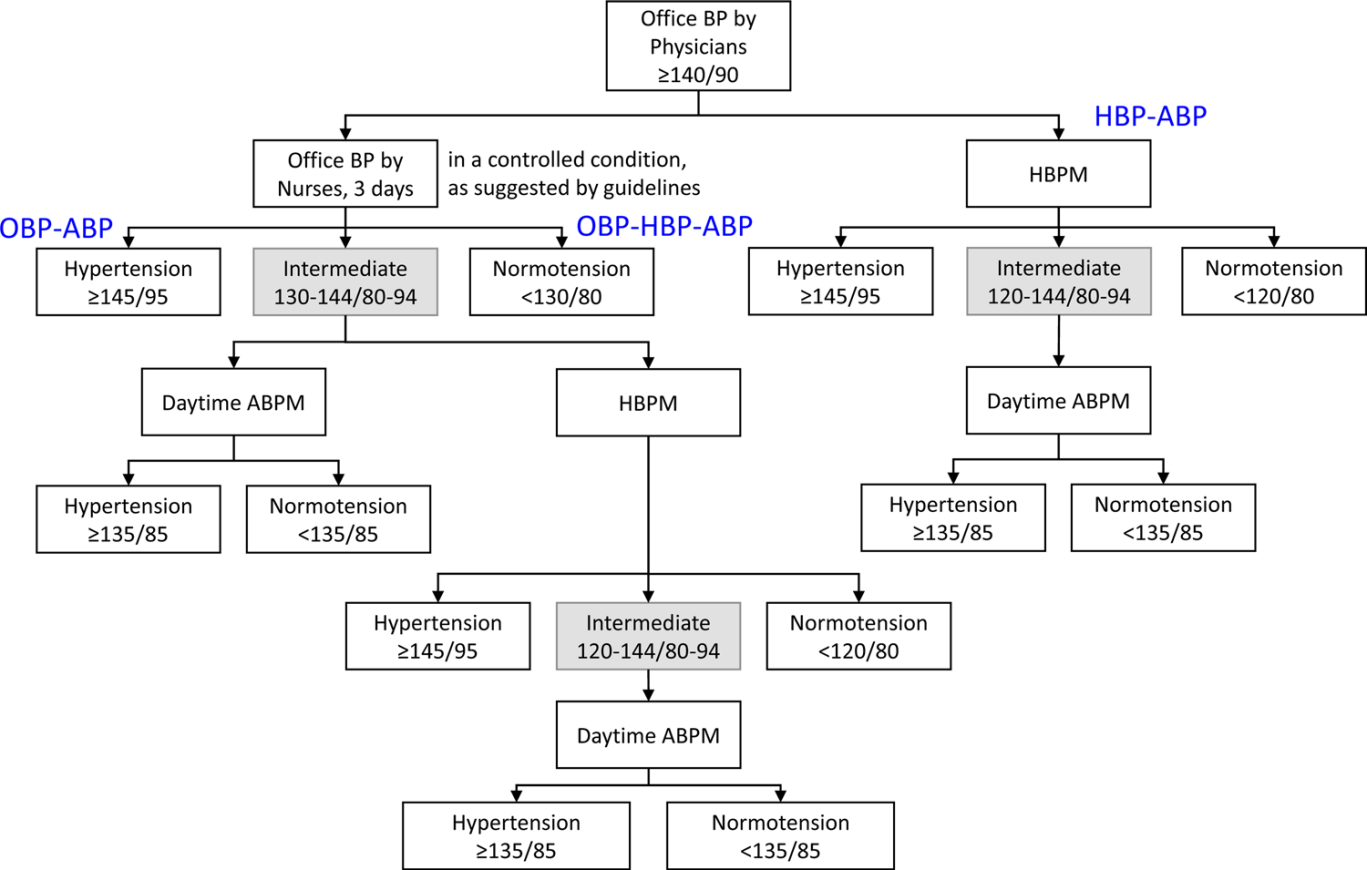
Algorithm for the diagnosis of hypertension using out‐of‐office blood pressure measurements. ABPM: ambulatory blood pressure measurements, HBPM: home blood pressure measurements, BP: blood pressure, OBP‐ABP: diagnosis of hypertension by office BP measurements for three visits and additional ambulatory BP measurements in the intermediate zone of office BP, OBP‐HBP‐ABP: diagnosis of hypertension by office BP measurements for three days, additional home BP measurement in the intermediate zone of office BP, and additional ambulatory BP measurements in the intermediate zone of home BP, HBP‐ABP: diagnosis of hypertension by home BP measurements and additional ambulatory BP measurement in the intermediate zone of home BP

In this study, we developed algorithms using out‐of‐office BP measurement for the diagnosis of hypertension (Figure 3). We introduced the intermediate zone showing a high frequency of mask hypertension and white‐coat hypertension. Exclusion of the intermediate zone revealed > 90% of the diagnostic sensitivity, PPV, and accuracy of home and office BP‐based diagnosis of hypertension. The diagnostic algorithm applying ambulatory BP or home BP measurement to the intermediate zone showed a diagnostic accuracy of hypertension more than 93%.

Discrimination of white‐coat hypertension from sustained hypertension is crucial to avoid unnecessary antihypertensive medication. Also, it is crucial to identify masked hypertension due to the similar or greater cardiovascular risk, compared to sustained hypertension.^20^, ^21^ Several studies and recent guidelines provided diagnostic algorithm using out‐of‐office BP measurements (home and ambulatory BP measurements) to determine white coat and masked hypertension in the diagnosis of hypertension.^5^, ^12^, ^13^, ^14^, ^15^, ^16^, ^22^, ^23^ Some focused on detecting white‐coat hypertension, not on masked hypertension. However, the high prevalence of masked hypertension and its prognostic implication involving adverse cardiovascular outcomes necessitated the inclusion of masked hypertension to the diagnostic algorithm for hypertension.^5^ The Predicting Out‐of‐Office Blood Pressure in the Clinic (PROOF‐BP) study^21^ provided an intermediate zone (130/80–144/90 mm Hg) for office BP at which patients are most likely to display white coat or masked hypertension. However, PROOF‐BP study did not include home BP measurements in the algorithm. Likewise, in 2018, the European Society of Cardiology and the European Society of Hypertension (ESC/ESH) guidelines for the management of arterial hypertension recommended the use of out‐of‐office BP (home or ambulatory BP) measurement in individuals with high‐normal BP (130–139/85–89 mm Hg) to detect masked hypertension but did not provide an upper threshold BP to detect white‐coat hypertension.^5^ Except for Mansoor and coworkers,^22^ no study provided the intermediate zone of home BP. Mansoor and coworkers recommended an intermediate zone of home BP where ambulatory BP should be measured. Although they did not use the term of masked hypertension, their algorithm introduced the lower home BP threshold to identify individuals with normal home BP who showed ambulatory hypertension.

Different from the 2018 ESH/ESC hypertension guidelines and previous studies, we have introduced office BP measured by using a standardized technique in the various guidelines and home BP measurement as a screening test and an intermediate zone of office and home BP. In terms of a reference standard method for the diagnosis of hypertension, current guidelines recommend both home and ambulatory BP measurements to detect white coat and masked hypertension, but it is left to the clinician's discretion which method is used for confirmation.^5^ Unfortunately, home BP and ambulatory BP measurements are not interchangeable.^10^, ^11^ Although current guidelines placed the same weight on home and ambulatory BP measurement in the diagnosis of hypertension, ambulatory BP measurement is considered as a reference standard for diagnosis of hypertension because there are fewer studies that link home BP monitoring to cardiovascular outcomes or mortality when compared to ambulatory BP measurements.^24^ Therefore, we used ambulatory BP measurement as the reference standard in the diagnosis of hypertension. Nevertheless, home BP has many advantages over ambulatory BP in that it is cheaper and more widely available and provides day‐to‐day BP variability (longitudinal monitoring). In this regard, we introduced the home BP arm as another screening test instead of using it as a confirmation test in our diagnostic algorithm for hypertension. Our algorithm offers physicians the choice of using home or ambulatory BP measurement in the diagnosis of hypertension.

The BP range of home BP intermediate zone in our study is wider than that in Mansoor and coworkers^22^ (120–144 mm Hg vs. 125–135 mm Hg and 80–94 mm Hg vs. 80–94 mm Hg, respectively). Although not being fully explained, it may be by the difference of sensitivity and specificity thresholds used in the ROC curve analysis (80% in Mansoor and coworkers and 95% in our study), as well as the small sample size of Mansoor and coworkers In general, it is estimated that BP measured at home is about 5 mm Hg lower than BP measured in the office. Reflecting this, the lower and upper thresholds of an intermediate zone in home SBP should be 125 mm Hg and 139 mm Hg (5 mm Hg lower than 130 mm Hg and 144 mm Hg for the office BP). However, in this study, those were determined at 120 mm Hg and 144 mm Hg for home SBP by the ROC curve method. Consequently, the intermediate zone of home SBP was 10 mm Hg broader than that of office SBP. Although the reason cannot be explained because there is no research on this phenomenon so far, the diagnostic disagreement between home and ambulatory BP measurement could be an explanation. There is a diagnostic disagreement between home and ambulatory BP in the diagnosis of hypertension, which is not uncommon.^10^, ^11^ In our validation population, the rate of disagreement was 21.1%.^11^ In the population showing diagnostic disagreement, 79.8% had ambulatory BP hypertension but home BP normotension. The reason for the diagnostic disagreement between ambulatory and home BP is unclear, but it may account for the wide range of home BP in the intermediate zone of our study. The BP range of office BP intermediate zone in our study cannot be directly compared to that of the PROOF‐BP study because the office BP in the PROOF‐BP study was calculated by a regression model.^23^

The advantages of our algorithm are (1) introduction of the intermediate zone, and (2) selection of office BP or home BP by physicians depending on the conditions. The inclusion of the majority of white‐coat and masked hypertension in the intermediate zone and application of ambulatory BP measurement may facilitate a more accurate diagnosis of hypertension phenotypes. As we describe in the above discussion, detection of white coat and masked hypertension is crucial to avoid unnecessary administration of antihypertensive medication and to prevent omission of high‐risk patients. The National Institute for Health and Care Excellence (NICE) guidelines recommend ambulatory or home BP measurements for patients when office BP is 140/90–179/119 mm Hg.^17^ However, ambulatory BP measurements in all the individuals with high office BP is not practical considering the limited availability of ambulatory BP measurement devices, high costs, and inconvenience. Ambulatory BP measurements only in the intermediate zone could reduce cost and inconvenience. Our algorithm can reduce the application of ambulatory BP measurement by ∼50% using the OBP‐ABP method, 60% for the OBP‐HBP‐ABP method, and 40% with the HBP‐ABP method. In clinical practice, frequent visits and controlled measurement of office BP can be burdensome. Instead of visits to measure office BP (OBP‐ABP and OBP‐HBP‐ABP strategy), HBP‐ABP strategy can reduce the number of clinic visits. Our algorithm allows the physicians to select an appropriate strategy based on the prevailing conditions at clinics and the patient's individual conditions.

There are limitations to this study. First, we determined daytime ambulatory BP as confirming diagnosis, rather than 24‐h ambulatory BP. Daytime ambulatory BP does not reflect nighttime BP. However, daytime ambulatory BP has the advantage of more effectively reflecting real office and home BP during the waking period while having the same diagnostic threshold as home BP in current guidelines.^5^ Second, office BP in our study was measured using the standardized technique recommended in the hypertension guidelines,^5^, ^6^ which may be different from those measured by a physician in routine clinical practice. Therefore, education regarding standardized office BP measurement techniques for healthcare providers may be required. The advantage of our algorithm is that we provided an alternative method to use home BP measurements instead of office BP measurements in controlled condition.

In conclusions, we proposed a diagnostic algorithm for hypertension using the intermediate zone of office BP, home BP, and both, followed by confirmation with ambulatory BP measurements. This algorithm may have an advantage in efficacy for the detection of masked and white coat hypertension.

## FUNDING

The study of development population was funded by Dong‐A ST Co. Ltd., Seoul, Korea. The sponsor was not involved in study design, study conduction, data interpretation, and the writing of the manuscript. The study of validation population was not funded.

## CONFLICTS OF INTEREST

The authors declare no conflicts of interest for the present study. MY Rhee has received lecture honoraria from Pfizer Inc., LG Life Sciences Ltd., Boehringer Ingelheim Pharma GmbH & Co. KG., Hanmi Pharm. Co. Ltd., Yuhan Co. Ltd., and Boryung Pharmaceutical Co. Ltd.; consulting fees from Hanmi Pharm. Co. Ltd. and Shin Poong Pharma. Co. Ltd.; and research grants from Boryung Pharmaceutical Co. Ltd. and Dong‐A Pharmaceutical Co. Ltd.

## AUTHOR CONTRIBUTIONS

All authors have read the manuscript and have approved this manuscript. All authors made substantial contributions to conception and design, acquisition of data, or analysis and interpretation of data; took part in drafting the review paper or revising it critically for important intellectual content; agreed on the journal to which the review paper will be submitted; gave final approval of the version of the review paper; and agreed to take responsibility and be accountable for all aspects of the work.

Moo‐Yong Rhee: Conceptualization, Methodology, Data curation, Formal analysis, Funding acquisition, Investigation, Writing‐ Original draft preparation. Je Sang Kim: Formal analysis, Writing‐ Original draft preparation. Chee Hae Kim, Yoo Ri Kim, Ungjeong Do, Ji‐Hyun Kim, Young Kwon Kim, Hyun Jung Lee, Jee Yeon Park: Writing ‐ Review & Editing. Ji‐Hyun Kim, June Namgung, Sung Yun Lee, Deok‐Kyu Cho, Tae‐Young Choi, and Seok Yeon Kim: Investigation, Writing ‐ Review & Editing.

## Supporting information

Supporting information.
