# Supplementary material for: Algorithm for diagnosing hypertension using out‐of‐office blood pressure measurements
Source: J Clin Hypertens (Greenwich). 2021 Oct 26;23(11):1965–74. doi: 10.1111/jch.14382 (PMC8630611; doi:10.1111/jch.14382)
Supplement: Supplementary file 1 — Supporting information. [file JCH-23-1965-s001.doc]

**Table S1. Diagnostic accuracy measures**

| **Measures** | **Formula** |
| --- | --- |
| Sensitivity | TP/(TP + FN) |
| Specificity | TN/(TN + FP) |
| Positive predictive value | TP/(TP + FP) |
| Negative predictive value | TN/(TN + FN) |
| Accuracy | (TP + TN)/(TP + FP + TN + FN) |
|  |  |
| **Definitions** |  |
| OBP-ABP arm |  |
| TP | TP=Number of subjects (ambulatory hypertension) in office hypertension + in office intermediate zone |
| TN | TN=Number of subjects (ambulatory normotension) in office normotension + in office intermediate zone |
| FN | FN=Number of subjects (ambulatory hypertension) in office normotension zone |
| FP | FP=Number of subjects (ambulatory normotension) in office hypertension zone |
|  |  |
| OBP-HBP-ABP arm |  |
| TP | TP=Number of subjects (ambulatory hypertension) in office hypertension zone + in home hypertension zone + in home intermediate zone |
| TN | TN=Number of subjects (ambulatory normotension) in office normotension + in home normotension zone + in home intermediate zone |
| FN | FN=Number of subjects (ambulatory hypertension) in office normotension zone + in home normotension zone |
| FP | FP=Number of subjects (ambulatory normotension) in office hypertension zone + in home hypertension zone |
|  |  |
| HBP-ABP arm |  |
| TP | TP=Number of subjects (ambulatory hypertension) in home hypertension + in home intermediate zone |
| TN | TN=Number of subjects (ambulatory normotension) in home normotension + in home intermediate zone |
| FN | FN=Number of subjects (ambulatory hypertension) in home normotension zone |
| FP | FP=Number of subjects (ambulatory normotension) in home hypertension zone |

TP: true positive, TN: true negative, FN: false negative, FP: false positive

**Table S2. Distribution of hypertension phenotypes according to zones of office and home blood pressure, referenced to 24-hour ambulatory blood pressure**

|  | Office BP | | |  | Home BP | | |
| --- | --- | --- | --- | --- | --- | --- | --- |
|  | Normotension | Intermediate | Hypertension |  | Normotension | Intermediate | Hypertension |
| Development |  |  |  |  |  |  |  |
| NT (n=48) | 13 (32.5) | 27 (67.5) | 0 (0.0) |  | 17 (42.5) | 22 (55.0) | 1 (2.5) |
| WH (n=20) | 0 (0.0) | 7 (77.8) | 2 (22.2) |  | 3 (33.3) | 6 (66.6) | 0 (0.0) |
| MH (n=42) | (3 (5.8) | 47 (90.4) | 2 (3.8) |  | 8 (15.4) | 42 (80.8) | 2(3.8) |
| SH (n=146) | 0 (0.0) | 44 (28.4) | 111 (71.6) |  | 5 (3.2) | 77 (49.7) | 73 (47.1) |
|  |  |  |  |  |  |  |  |
| Validation |  |  |  |  |  |  |  |
| NT (n=47) | 13 (31.0) | 29 (69.0) | 0 (0.0) |  | 8 (18.6) | 35 (81.4) | 0 (0.0) |
| WH (n=26) | 0 (0.0) | 18 (78.3) | 5 (21.7) |  | 2 (9.1) | 20 (90.9) | 0 (0.0) |
| MH (n=63) | 3 (4.5) | 63 (95.5) | 0 (0.0) |  | 7 (10.4) | 55 (82.1) | 5 (7.5) |
| SH (n=263) | 0 (0.0) | 88 (32.8) | 180 (67.2) |  | 3 (1.1) | 152 (56.9) | 112 (41.9) |

Hypertension phenotypes in office BP and home BP were defined by office BP hypertension (≥140/90 mmHg) and ambulatory BP hypertension (≥135/85 mmHg), and home BP hypertension (≥135/85 mmHg) and ambulatory daytime BP hypertension (≥135/85 mmHg), respectively

BP: blood pressure, NT: normotension, WH: white-coat hypertension, MH: masked hypertension, SH: sustained hypertension

**Table S3. Diagnostic sensitivity and specificity of office BP and home BP based diagnosis of hypertension, referenced to 24-hour ambulatory blood pressure**

|  | development | | | | | Validation | | | | |
| --- | --- | --- | --- | --- | --- | --- | --- | --- | --- | --- |
|  | Sensitivity, %  (95%CI) | Specificity, %  (95%CI) | PPV, %  (95%CI) | NPV, %  (95%CI) | Accuracy % (95%CI) | Sensitivity, %  (95%CI) | Specificity, %  (95%CI) | PPV, %  (95%CI) | NPV, %  (95%CI) | Accuracy % (95%CI) |
| All |  |  |  |  |  |  |  |  |  |  |
| Office BP | 75.6  (69.1 – 81.3) | 78.4  (64.7 – 88.7) | 93.4  (89.2 – 96.0) | 44.4  (37.7 – 51.4) | 76.2  (70.5-81.3) | 71.8  (65.6 – 77.5) | 64.6  (51.8 – 76.1) | 88.0  (83.9 – 91.1) | 38.9  (32.6 – 45.5) | 70.2  (64.7 – 75.4) |
| Home BP | 77.1  (70.7 – 82.6) | 78.4  (64.7 – 88.7) | 93.5  (89.4 – 96.1) | 46.0  (38.9 – 53.2) | 77.3  (71.7-82.3) | 78.4  (73.6 – 82.7) | 80.0  (68.2 – 88.9) | 95.3  (92.5 – 97.0) | 41.9  (36.3 – 47.8) | 78.7  (74.3 – 82.6) |
| Excluding intermediate zone |  |  |  |  |  |  |  |  |  |  |
| Office BP | 97.4  (92.5 – 99.5) | 76.5  (50.1 – 93.2) | 96.5  (92.2 – 98.5) | 81.3  (57.9 – 93.2) | 94.7  (89.3-97.8) | 98.4  (95.3 – 99.7) | 72.2  (46.5 – 90.3) | 97.3  (94.5 – 98.7) | 81.3  (57.6 – 93.2) | 96.0  (92.3 – 98.3) |
| Home BP | 90.5  (84.0 – 95.0) | 77.8  (57.7 – 91.4) | 95.0  (90.3 – 97.5) | 63.6  (49.6 – 75.7) | 88.2  (82.0-92.9) | 95.1  (91.1 – 97.6) | 76.9  (46.2 – 95.0) | 98.5  (96.0 – 99.4) | 50.0  (33.8 – 66.2) | 94.0  (89.9 – 96.8) |

Non-overlapping of 95% confidence intervals indicates statistical difference.

PPV: positive predictive value, NPV: negative predictive value, CI: confidence interval, BP: blood pressure

**Table S4. Diagnostic accuracy, application rate of ABP, and summation of visiting counts in the study population, referenced to 24-hour ambulatory blood pressure**

|  | Development | | |  | Validation | | |
| --- | --- | --- | --- | --- | --- | --- | --- |
|  | OBP-ABP | OBP-HBP-ABP | HBP-ABP |  | OBP-ABP | OBP-HBP-ABP | HBP-ABP |
| Sensitivity, % (95%CI) | 98.5  (95.8 – 99.7) | 94.6  (90.6 – 97.3) | 94.1  (90.0 – 96.9) |  | 99.1  (97.4 – 99.8) | 97.0  (94.6 – 98.6) | 97.0  (94.6 – 98.6) |
| Specificity, % (95%CI) | 92.2  (81.1 – 97.8) | 84.3  (71.4 – 93.0) | 88.2  (76.1 – 95.6) |  | 92.3  (83.0 – 97.5) | 89.2  (79.1 – 95.6) | 95.4  (87.1 – 99.0) |
| AUC  (95%CI) | 0.953  (0.920 – 0.976) | 0.895  (0.851 – 0.930) | 0.912  (0.870 – 0.944) |  | 0.957  (0.932 – 0.975) | 0.931  (0.902 – 0.954) | 0.962  (0.938 – 0.978) |
| PPV, % (95%CI) | 98.1  (95.2 – 99.2) | 96.0  (92.8 – 97.9) | 97.0  (93.8 – 98.6) |  | 98.5  (96.6 – 99.4) | 97.9  (95.8 – 98.9) | 99.1  (97.3 – 99.7) |
| NPV, % (95%CI) | 94.0  (83.6 – 98.0) | 79.6  (68.5 – 87.5) | 78.9  (68.2 – 86.8) |  | 95.2  (86.6 – 98.4) | 85.3  (75.8 – 91.5) | 86.1  (77.1 – 92.0) |
| Accuracy, % (95%CI) | 97.3  (94.4 – 98.9) | 92.6  (88.7 – 95.5) | 93.0  (89.1 – 95.8) |  | 98.0  (96.1 – 99.1) | 95.7  (93.3 – 97.5) | 96.7  (94.5 – 98.3) |

Non-overlapping of 95% confidence intervals indicates statistical difference.

AUC: Area under the curve, PPV: positive predictive value, NPV: negative predictive value, CI: confidence interval


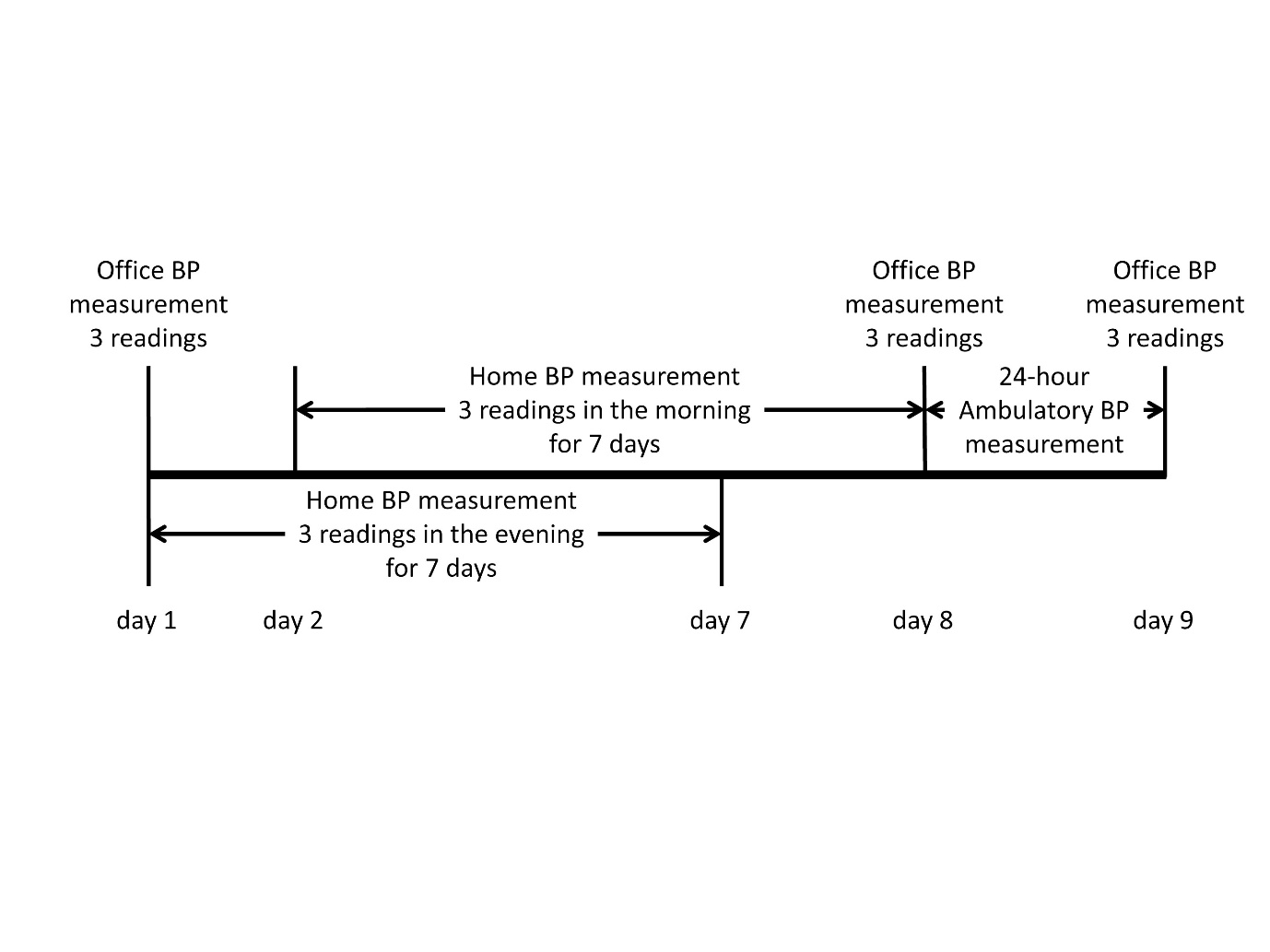


Figure S1. Schedule of blood pressure measurement. BP, blood pressure


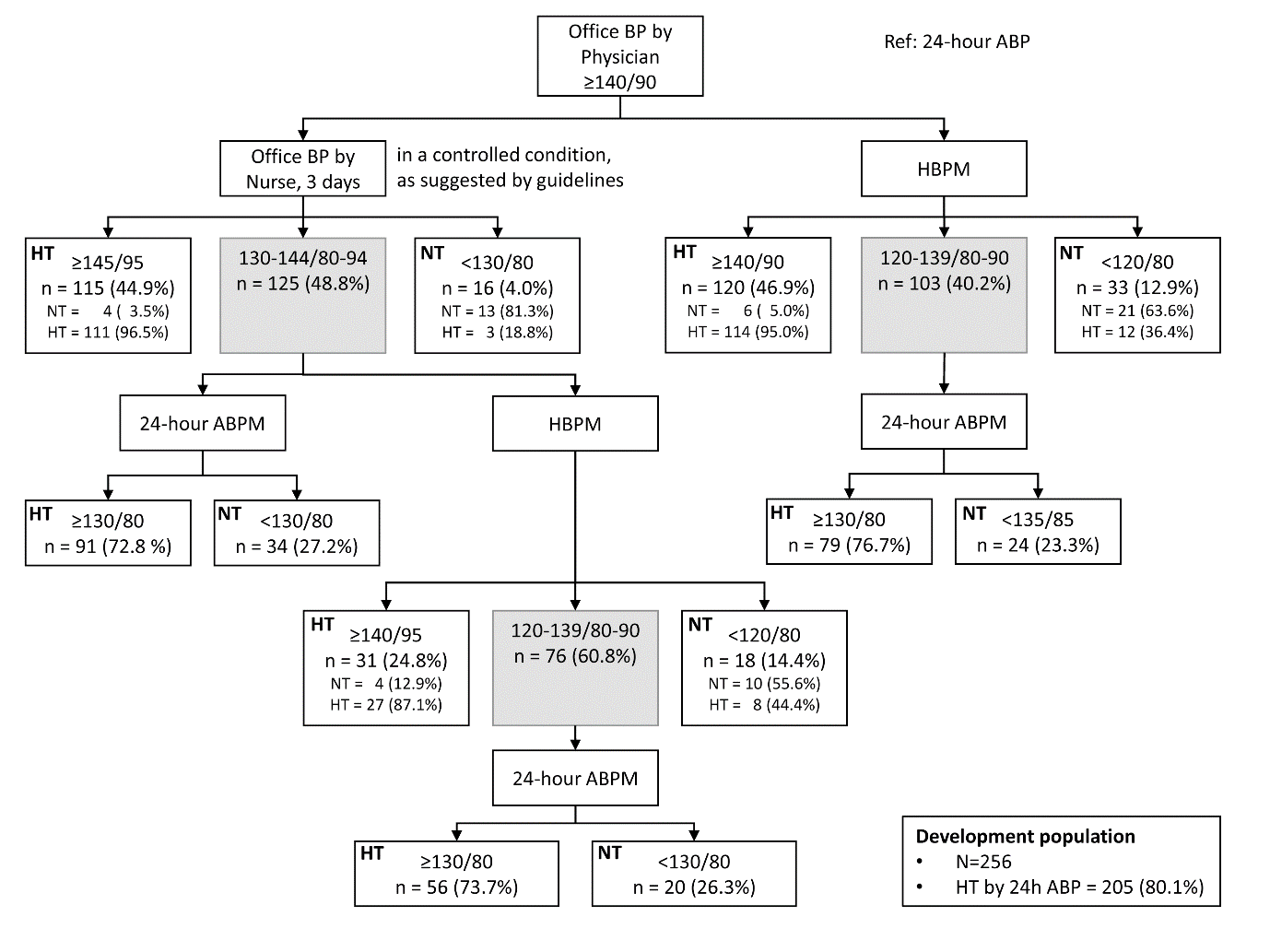


Figure S2. Distribution of participants according to level of office blood pressure, home blood pressure and ambulatory 24-hour blood pressure in the development population. BP, blood pressure; HT, hypertension; NT, normotension; ABP, ambulatory blood pressure; ABPM, ambulatory blood pressure measurements; HBPM, home blood pressure measurements.


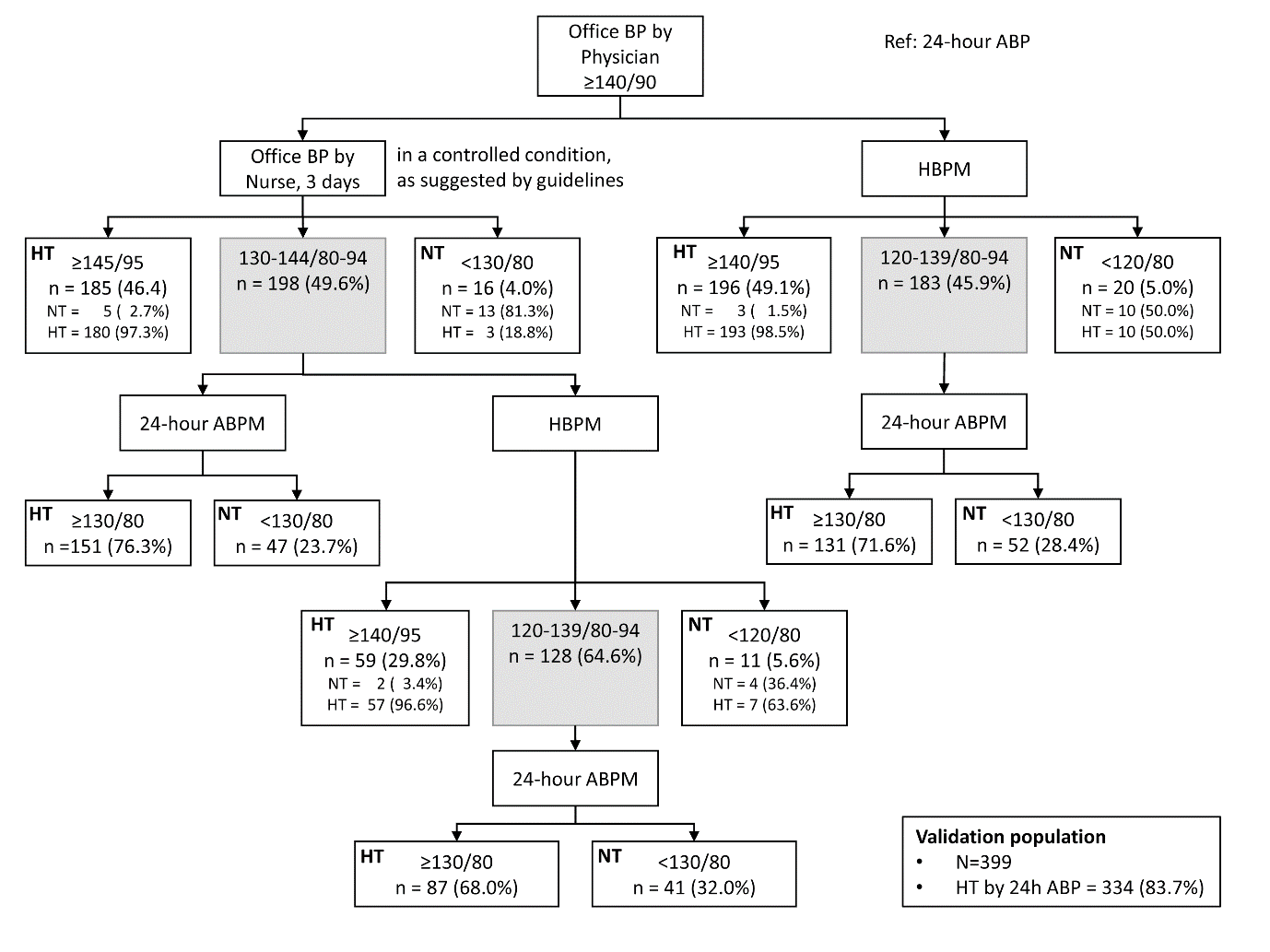


Figure S3. Distribution of participants according to level of office blood pressure, home blood pressure and ambulatory 24-hour blood pressure in the validation population. BP, blood pressure; HT, hypertension; NT, normotension; ABP, ambulatory blood pressure; ABPM, ambulatory blood pressure measurements; HBPM, home blood pressure measurements.
